# Beyond Homologous Recombination: Functional Modules of Olaparib Resistance Revealed by CRISPR Screens

**DOI:** 10.3390/ijms27209381

**Published:** 2026-10-21

**Authors:** Dana Dildabayeva, Diana Samarkhanova, Larissa Lezina

**Affiliations:** Department of Biological and Biomedical Sciences, School of Medicine, Nazarbayev University, Astana 010000, Kazakhstan; dana.dildabayeva@nu.edu.kz (D.D.); diana.samarkhanova@nu.edu.kz (D.S.)

**Keywords:** olaparib, PARP inhibitor resistance, CRISPR screen, functional genomics

## Abstract

Clinical use of PARP inhibitors (PARPi), including olaparib, has improved outcomes for patients with BRCA1/2-mutant and homologous recombination (HR)-deficient tumors. However, acquired resistance remains a major limitation to their efficacy. CRISPR-Cas9 screening approaches have been developed to identify genetic drivers of PARPi resistance. However, differences in experimental conditions, library coverage, and selection design complicate comparisons across studies. In this review, we present a harmonized reanalysis of CRISPR knockout olaparib resistance screens to identify recurrent resistance-associated genes and pathways. Datasets with raw data were collected from the BioGRID ORCS database. Resistance hits identified in at least two screens were subjected to Reactome pathway-enrichment analysis and organized into functional modules. Using this approach, we identified eight modules: DNA-damage repair and replication stress; cell-cycle regulation; chromatin remodeling and histone modification; post-translational regulation; mitochondrial function; TGF-β and mTOR signaling; RNA processing and nucleocytoplasmic transport; and emerging adaptive mechanisms. Importantly, mitochondrial function genes were observed as recurrent hits, but their relation to olaparib resistance is underinvestigated. Our integrative analysis suggests that PARPi resistance is a complex, adaptive phenotype shaped by recurrent but context-dependent vulnerabilities across multiple cellular programs. This framework prioritizes candidate resistance drivers for validation and highlights directions for the design of combination therapies aimed at overcoming PARPi resistance.

## 1. Introduction

PARP inhibitors have transformed the treatment of *BRCA1/2*-mutant and other homologous-recombination-deficient cancers. However, their clinical benefit is frequently limited by the emergence of resistance. Understanding how tumor cells evade PARP inhibition requires both a clear picture of the drug’s mechanism of action and a systematic view of the genetic routes to resistance. In this review paper, we discuss mechanisms of PARP inhibitor action and functional pathways through which resistance may arise, with particular attention to the insights coming from CRISPR-KO screening studies.

### 1.1. PARP Inhibitors: Mechanism and Clinical Application

Poly(ADP-ribose) polymerases (PARPs) catalyze the post-translational modification of proteins by transferring ADP-ribose moieties from NAD^+^ [1]. PARP1 and PARP2 recognize DNA single-strand breaks (SSBs) and single-strand gaps, undergo auto-PARylation, and recruit XRCC1 together with other components of the base excision repair (BER) machinery [2]. Clinically approved PARP inhibitors (PARPi), including olaparib, rucaparib, niraparib, and talazoparib, are used in the treatment of ovarian, breast, pancreatic, and prostate cancers. Their therapeutic rationale is based on synthetic lethality, whereby cells with homologous recombination deficiency (HRD) are particularly sensitive to PARP inhibition [3,4]. PARPi suppresses PARP catalytic activity and can trap PARP1 on DNA, generating nucleoprotein complexes that impede replication-fork progression and create lesions that require homologous recombination (HR) for efficient resolution [5].

PARP-trapping capacity varies among PARPi and contributes to differences in their cytotoxic potency. Talazoparib is the most potent PARP trapper, whereas niraparib, olaparib, and rucaparib exhibit intermediate trapping activity. Interestingly, another potent investigational PARPi, veliparib, has relatively weak trapping capacity [6]. Although talazoparib exhibits the greatest PARP-trapping potency among clinically used PARP inhibitors, it is not the most effective therapeutic. This indicates that differences in trapping potency do not directly translate into a consistent hierarchy of clinical efficacy, which varies according to tumor type, molecular context, and treatment setting. Persistent single-stranded DNA (ssDNA) gaps also contribute to PARPi sensitivity in HRD cells and are reduced as resistance develops [7].

### 1.2. The Clinical Challenge: Resistance to PARP Inhibitors

The clinical success of PARPi in treating BRCA1/2-mutated and HR-deficient cancers is one of the significant achievements in precision oncology. However, despite high initial response rates, both intrinsic and acquired resistance have emerged as a major limitation of the treatment [8].

Several molecular mechanisms of PARPi resistance are already well established, including restoration of homologous recombination (HR), rewiring of DNA-damage response pathways, and stabilization of replication forks [9]. Among mechanisms that restore HR, secondary reversion mutations in *BRCA1* or *BRCA2* are the best characterized. These mutations restore the open reading frame and can produce partially functional BRCA proteins, thereby reducing dependence on PARP-mediated repair and overcoming PARPi-induced synthetic lethality [10].

Another mechanism of HR restoration involves loss of 53BP1 or its downstream effectors, including RIF1 and the Shieldin complex. This relieves the restriction on DNA-end resection and shifts double-strand break repair toward HR. This mechanism is particularly relevant in *BRCA1*-deficient tumors, where increased end resection can partially restore HR capacity despite continued loss of BRCA1 function [11].

Other mechanisms of PARPi resistance bypass HR restoration altogether. Upregulation of drug-efflux transporters, particularly ABCB1 (P-glycoprotein/MDR1), can reduce intracellular PARPi concentrations independently of DNA-repair status [12]. Loss of PARP1 itself can also confer resistance by removing the molecular target required for PARP trapping on DNA [13]. In addition, stabilization of stalled replication forks can protect cells from PARPi-induced replication stress. This may occur through loss of RADX or SLFN11, or through restoration of RAD51 filament formation. Notably, RADX loss appears to preferentially restore fork protection in *BRCA2*-deficient rather than *BRCA1*-deficient cells [14].

### 1.3. Approaches to Studying Drug Response and the Emergence of CRISPR Screening

Drug resistance and sensitivity can be investigated using several approaches: candidate-gene studies, in vitro resistance evolution, analysis of clinical samples, and functional genetic screens. Candidate-gene approaches test hypotheses based on established biology but are poorly suited for discovering unexpected resistance factors. In in vitro resistance-evolution models, sensitive cell lines are exposed to progressively increasing drug concentrations over extended periods. Resistant derivatives are then subjected to genomic or molecular analysis. This approach can reveal mechanisms of acquired resistance but is time-consuming and highly model-dependent [15,16,17].

Clinical samples provide the most direct insight into resistance arising in patients. These studies may include paired pre- and post-treatment biopsies, serial circulating tumor DNA (ctDNA), and pathological analysis of tumors obtained after disease progression [18]. However, their scope is limited by sample availability and by spatial and temporal tumor heterogeneity.

Functional genetic screens provide a complementary, unbiased approach. They allow systematic testing of thousands of genes for their contribution to drug sensitivity or resistance. Before the development of CRISPR, mammalian genetic screens relied mainly on RNA interference (RNAi) using short hairpin RNA (shRNA) or small interfering RNA (siRNA) libraries. Although productive, these approaches are limited by incomplete gene suppression, seed-dependent off-target effects, and variable reproducibility. Genome-scale CRISPR-Cas9 libraries, introduced in 2014, enabled more robust loss-of-function screening [19,20]. In pooled screens, CRISPR knockout subsequently showed greater performance for identifying essential genes than either shRNA or CRISPR interference (CRISPRi) approaches [21].

The value of such unbiased genetic approaches is illustrated by the identification and subsequent refinement of the 53BP1 pathway as a major determinant of PARPi response. Loss of 53BP1 in *BRCA1*-deficient cells restores DNA-end resection and partially restores HR, thereby promoting PARPi resistance [22]. Subsequent CRISPR-Cas9 screens expanded this mechanism by identifying additional components of the 53BP1 pathway involved in resistance [13,23].

### 1.4. The Methodological Evolution: Toward CRISPR-Cas9 Screening

As noted above, CRISPR-based screens are particularly well suited to studying PARPi resistance because they introduce stable and highly specific genetic perturbations across large numbers of genes. In brief, genome-wide CRISPR-Cas9 screening is a functional genomics approach that systematically evaluates gene function across the genome. A pooled library of single-guide RNAs (sgRNAs) is introduced into Cas9-expressing cells, typically so that each cell receives one dominant genetic perturbation. Multiple sgRNAs are usually designed to target each gene, improving the robustness and efficiency of gene targeting. Following drug treatment, sgRNA abundance is quantified by sequencing and compared with an untreated or baseline reference population to identify genes associated with altered drug response [24].

CRISPR screens can be broadly divided into CRISPR knockout (CRISPR-KO), CRISPR activation (CRISPRa), and CRISPR interference (CRISPRi). CRISPR-KO uses Cas9-induced double-strand breaks to generate loss-of-function mutations and is widely used to identify genes whose disruption alters drug sensitivity or resistance. In contrast, CRISPRa and CRISPRi use catalytically inactive dCas9 to modulate transcription without changing the underlying DNA sequence. CRISPRa increases target-gene expression and is useful for identifying gain-of-function resistance mechanisms, whereas CRISPRi represses transcription and is particularly useful for studying essential genes that cannot be fully knocked out [25].

Screen design also depends on whether the goal is to identify targets responsible for resistance or sensitivity. In resistance screens, cells are exposed to selective drug pressure, and sgRNAs enriched in surviving cells indicate gene perturbations that confer a fitness advantage. In sensitivity screens, sgRNAs that become depleted under treatment identify genes whose loss reduces fitness or increases drug susceptibility. The outcome of either screen is strongly influenced by experimental variables, including cell line, drug concentration, treatment duration, and library design [26].

In this review, we reanalyze published olaparib CRISPR knockout screens using a standardized analytical workflow to prioritize candidate resistance genes identified in at least two screens. We discuss the recurrent hits in relation to published experimental findings. This comparison adds to existing reviews by helping to identify genes worth investigating further. The modules discussed below include established resistance determinants and candidates whose proposed mechanisms require validation.

## 2. Functional Modules of Olaparib Resistance Genes Revealed by CRISPR Screens

The present analysis examines olaparib-response CRISPR screens reported in ten published studies [27,28,29,30,31,32,33,34,35,36], including both genome-wide and targeted libraries. Although some studies also employed CRISPR interference (CRISPRi) or activation (CRISPRa), only CRISPR knockout (CRISPR-KO) data were included in the cross-screen analysis. Key experimental parameters, including cancer type, cell model, screening library, drug dose, and principal findings, are summarized in Table 1.

The integrated reanalysis included ten published studies comprising 19 study–cell-line screening units. Following the consolidation of repeated time points and complementary libraries, 163 genes were identified as positive-selection hits in at least two screening units (Supplementary Table S1). Of these, 109 genes were supported by screens reported in at least two publications, whereas 54 were identified in multiple cell-line screens within a single publication. This distinction was considered when interpreting recurrence as evidence of potential resistance-associated mechanisms.

**Figure 1:**
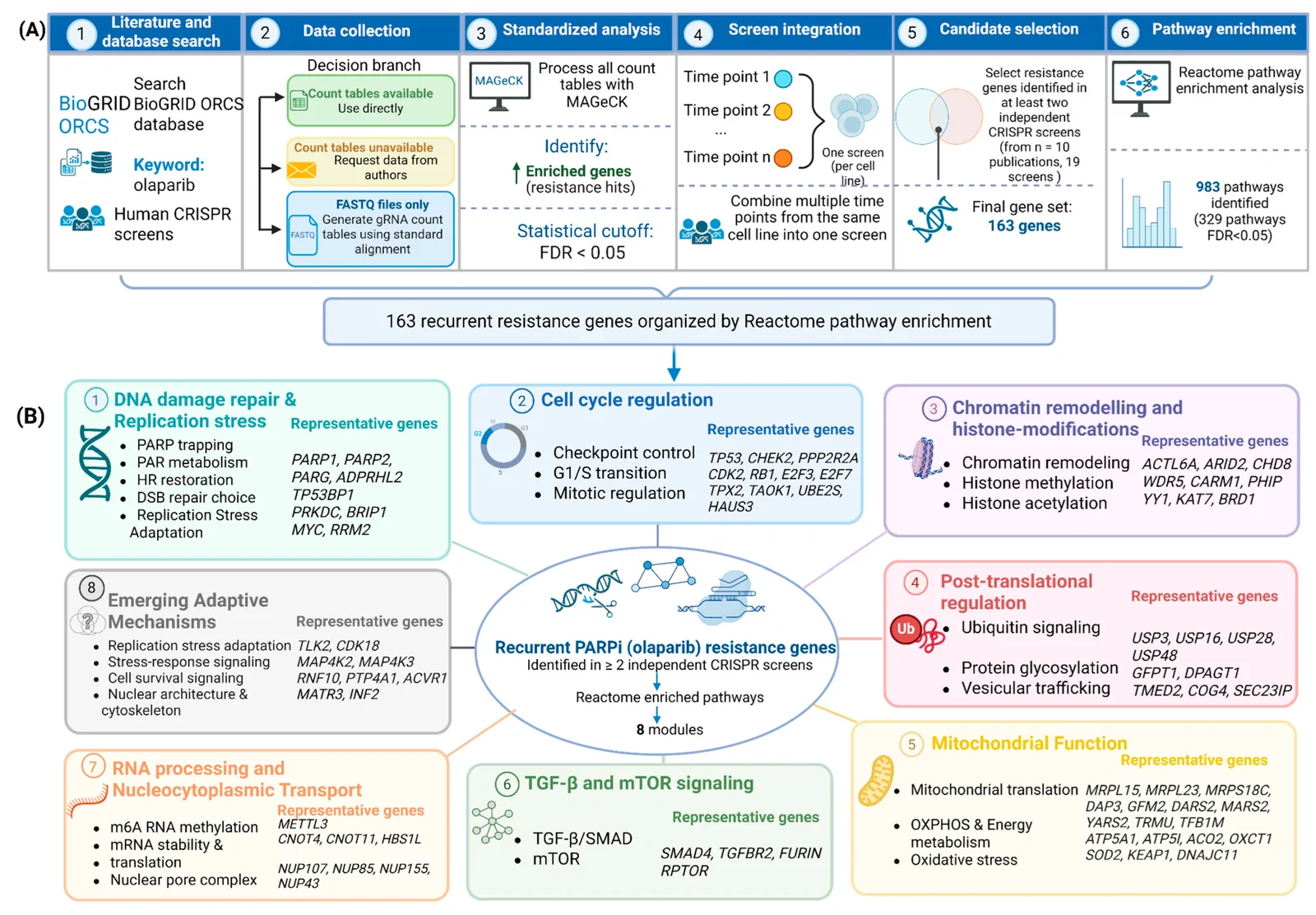
Systematic identification and functional classification of recurrent olaparib resistance genes from independent CRISPR-Cas9 screens. (**A**) Screening and analysis pipeline. Published CRISPR-Cas9 knockout screens involving olaparib were identified through the BioGRID Open Repository of CRISPR Screens (BioGRID ORCS). Gene-level count tables were obtained from public sources, original authors, or regenerated from raw FASTQ files. Datasets were reprocessed using MAGeCK, and resistance hits were defined as genes significantly enriched in olaparib-treated cells (FDR < 0.05). Multiple time points from the same cell line were combined into a single screen before recurrence analysis. Genes identified in at least two of nineteen independent screens yielded 163 recurrent resistance genes, followed by Reactome pathway enrichment analysis (329 significantly enriched pathways with FDR < 0.05) (Table S2). (**B**) Functional classification of recurrent resistance-associated genes into seven modules derived from significantly enriched Reactome pathways and an eighth, literature-informed group comprising genes not represented in these enriched pathways. Each gene was assigned to one primary functional module based on its literature-supported biological function. Complete gene-to-module assignments are provided in Supplementary Table S2. Colors in panel B are used to distinguish the eight functional modules and do not represent differences in statistical significance or strength of experimental evidence. Connecting lines indicate functional classification rather than experimentally validated molecular interactions. Abbreviations: CRISPR-Cas9, clustered regularly interspaced short palindromic repeats and CRISPR-associated protein 9; MAGeCK, Model-based Analysis of Genome-wide CRISPR-Cas9 Knockout; FDR, false discovery rate; TGF-β, transforming growth factor beta; mTOR, mechanistic target of rapamycin. (Created in BioRender. Lezina, L. (2026) https://BioRender.com/8gufd7a, accessed on 16 October 2026).

Reactome pathway-enrichment analysis of this gene set identified 329 statistically significant pathways out of 983, which were grouped into eight functional modules based on literature representing distinct biological mechanisms of PARPi resistance (Figure 1A and Table S2). These modules are summarized in Table 2 and Figure 1B, together with representative genes, proposed mechanisms, and the strength of supporting evidence. The modules range from well-established DNA-damage response pathways with strong independent validation to emerging metabolic and post-transcriptional adaptations supported primarily by screening data. The level of evidence for each module is indicated in Table 2 and discussed in the corresponding sections below.

### 2.1. DNA Damage Repair and Replication Stress

DNA-damage repair and replication-stress genes formed the most important functional category in our analysis. This resistance module includes the PARP enzymes themselves, regulators of PAR turnover, factors controlling double-strand break repair-pathway choice, and genes that set the overall replication burden. Olaparib is cytotoxic because it traps PARP1 on DNA, and trapped complexes are lethal only when homologous recombination cannot repair the break they generate. Resistance in this module therefore may arise through reduced trapping, through restored end resection, or through a lower rate of fork collision with trapped complexes.

#### 2.1.1. The PARP1–DNA Trapping Axis: PARP1, PARP2

PARP1 trapping on DNA is a central component of PARPi cytotoxicity. PARP1 recognizes single-strand breaks (SSBs) and promotes their repair through poly(ADP-ribose) synthesis and recruitment of DNA-repair factors. PARPi inhibits this catalytic activity and stabilizes PARP1–DNA complexes, generating cytotoxic replication-associated lesions. Collision of replication forks with trapped PARP1 can produce one-ended double-strand breaks (DSBs), contributing to cell death [37]. Consistent with this mechanism, PARP1 is among the most recurrent resistance hits across CRISPR-KO screens.

PARP2 has partially overlapping functions with PARP1 and also emerged as a recurrent hit, although its contribution to PARP trapping appears more limited than that of PARP1 [5]. A “tag–mutate–enrich” CRISPR mutagenesis approach further identified specific *PARP1* point mutations, particularly within the zinc-finger DNA-binding domains. These variants can retain partial enzymatic activity while reducing olaparib-induced PARP trapping. Related *PARP1* variants have also been reported in clinical cases of acquired PARPi resistance, supporting *PARP1* mutation as a bona fide resistance mechanism [13].

#### 2.1.2. PAR Metabolism as a Regulator of Trapping: PARG, ADPRHL2

CRISPR screens also identified the ADP-ribosylhydrolases PARG and ADPRHL2 (ARH3) as recurrent resistance-associated genes. Loss of either enzyme alters ADP-ribose turnover and can affect the stability of PARP1–DNA complexes [38,39].

Loss of PARG causes accumulation of PAR chains and can promote PARP1 auto-release from DNA, thereby reducing cytotoxic trapping independently of HR restoration. Loss of ADPRHL2 similarly alters ADP-ribose turnover and may destabilize the molecular state required for efficient drug-induced trapping [40,41].

#### 2.1.3. Restoration of Homologous Recombination: TP53BP1

In *BRCA1*-deficient cells, loss of *TP53BP1* relieves the block on DNA-end resection and can partially restore homologous recombination. This reduces synthetic lethality and thereby can promote PARPi resistance [42].

#### 2.1.4. Alternative DSB Repair and Repair-Pathway Choice: PRKDC, BRIP1

Alterations in DSB repair-pathway choice may also influence PARPi response. *PRKDC*, which encodes DNA-PKcs, is a central component of non-homologous end joining (NHEJ). Its loss can alter the balance between NHEJ and homologous recombination in a context-dependent manner and thereby modify PARPi sensitivity [43,44].

*BRIP1* (FANCJ), a DNA helicase that interacts with the BRCT domain of BRCA1, also emerged as a functional-screen hit. This finding suggests that altered helicase-dependent DNA processing may contribute to resistance in specific genetic contexts [45].

#### 2.1.5. Replication-Stress Adaptation and Cellular Fitness: MYC, RRM2

MYC is a potent driver of replication stress through accelerated S-phase progression and increased transcription–replication conflicts. Loss of MYC may therefore reduce the replication burden imposed on the cell. Fewer or slower replication forks could decrease the probability of collision with trapped PARP1 complexes and thereby may promote survival during PARPi treatment [46].

The interpretation of *RRM2* is less straightforward. RRM2 depletion or inhibition reduces dNTP availability and generally increases replication stress, and can sensitize cells to PARP inhibition [47]. However, RRM2 was identified as a hit in two prostate cancer cell-line screens included in our analysis. These contrasting observations suggest that the effect of RRM2 on PARP inhibitor response may be context-dependent.

### 2.2. Cell-Cycle Regulation

Cell-cycle genes, particularly those controlling S-phase entry and progression, were among the most represented functional categories in our analysis. This functional module includes checkpoint regulators, G1/S-transition factors, and mitotic genes. PARP1 trapping is especially toxic during the S phase because it promotes replication-fork stalling and double-strand break formation, whereas homologous recombination is largely restricted to S/G2. PARPi response may therefore depend strongly on cell-cycle progression, checkpoint integrity, and apoptotic competence [5,48].

#### 2.2.1. Checkpoint Control: TP53, CHEK2, and PPP2R2A

Cell-cycle checkpoints normally delay cell-cycle progression in response to DNA damage. Their disruption may permit continued proliferation despite unrepaired lesions and may reduce damage-induced apoptosis [49]. In prostate cancer models, CRISPR screens identified *TP53* and *CHEK2* as resistance-associated hits [33]. Loss of CHEK2 weakens p53-dependent checkpoint signaling and can reduce G1 arrest and apoptosis after DNA damage [33].

*PPP2R2A*, a regulatory subunit of PP2A, presents a more complex case. Its loss has been reported to hyperactivate ATM–CHK2 signaling, reduce BRCA1 and RAD51 expression, and impair homologous recombination. Consistent with this, *PPP2R2A* depletion sensitized NSCLC cells to PARPi [50]. *PPP2R2A* was included in the homologous recombination repair panel used in the PROfound prostate cancer study [50]. Clinically, however, patients with *PPP2R2A* alterations had poorer outcomes on olaparib [51]. CRISPR screens in prostate cancer models likewise identified *PPP2R2A* as a resistance-associated hit [33]. These discordant findings highlight the need for lineage-specific validation of candidate PARPi biomarkers.

#### 2.2.2. G1-to-S Transition: CDK2, RB1, E2F3, and E2F7

Alterations in G1/S regulators may modify PARPi response by changing the fraction of cells entering S phase and by reshaping DNA-repair capacity. Loss of *CDK2* may delay S-phase entry and reduce the number of replication forks exposed to trapped PARP1. However, this effect is context-dependent. In *BRCA1*-mutant models, CDK2 inhibition can increase DNA damage and synergize with PARPi [52]. However, in HR-proficient CRISPR screens *CDK2* loss has appeared as a resistance hit [52]. These opposing effects likely reflect differences in genetic background and perturbation context.

*RB1* is a critical regulator of cell cycle arrest in G1 phase. Its loss derepresses E2F1 and may increase expression of BRCA1, BRCA2, and RAD51, thereby enhancing HR capacity and reducing PARPi sensitivity [53]. In prostate cancer models, the effect of *RB1* loss on PARPi response depended on the surrounding genetic context. Loss of *RNASEH2B* sensitized cells to PARP inhibition, but concurrent *RB1* deletion largely abolished this sensitivity. Combined PARP1 and ATR inhibition suppressed E2F1-dependent BRCA1/2 and RAD51 expression, impaired HR, and resensitized *RNASEH2B/RB1*-deficient cells to PARP inhibition [53]. However, the effect of *RB1* loss on PARPi response is strongly context-dependent. In some prostate cancer models, *RB1* loss can reduce PARPi sensitivity by increasing E2F1 activity and enhancing expression of HR factors such as BRCA2. However, BRCA2 loss may override the reduced PARPi sensitivity associated with RB1 loss [53,54]. In *BRCA2/RB1*-co-deleted prostate cancer models, the profound HR defect caused by BRCA2 loss remains dominant, resulting in preserved or even increased sensitivity to PARPi compared with *BRCA2* loss alone. These findings suggest that the effect of RB1 loss on PARPi response may depend on the accompanying defects in HR [54].

*E2F7* depletion has been linked to olaparib resistance in *BRCA2*-deficient models, together with increased RAD51 expression and enhanced HR [55,56]. By contrast, *E2F3* knockdown sensitized breast cancer cells to olaparib, despite its resistance-associated enrichment in our integrated analysis [57]. These findings argue against a single shared G1/S-delay mechanism and suggest model-specific effects shaped by baseline proliferation and DNA-repair capacity.

#### 2.2.3. Mitotic Regulators: UBE2S, TAOK1, TPX2, and HAUS3

Our reanalysis also identified a mitotic sub-cluster containing *TAOK1, TPX2,* and *HAUS3*. These genes have been linked to mitotic control, DNA repair, or tolerance of replication-associated stress. TAOK1 suppression impaired USP7–RAD51-dependent HR and increased PARPi sensitivity [58]. TPX2 supports HR and replication-fork protection, and its depletion sensitized pancreatic cancer models to olaparib [59,60]. *UBE2S* was not identified in our reanalysis of screens, but is discussed here because experimental studies have linked it to olaparib response [61,62]. The effect of UBE2S appears context-dependent: its knockdown increased olaparib sensitivity in ovarian cancer cells [61], whereas in Cezanne-deficient models it restored BRCA1 levels and reversed PARPi hypersensitivity [62]. The resistance-associated effect of *HAUS3* loss remains insufficiently characterized and requires independent validation.

Overall, these findings suggest that cell-cycle genes can influence PARPi response through multiple mechanisms, including checkpoint attenuation, altered S-phase entry, changes in HR capacity, and tolerance of mitotic stress. Their effects are highly context-dependent and should not be reduced to a single cell-cycle-delay model.

### 2.3. Chromatin Remodeling and Histone Modification

In the context of PARPi, chromatin regulates the stability of entrapped PARP1 on the nucleosome and the access of the repair machinery to the lesion. Histone ADP-ribosylation facilitates PARP1 release from DNA lesions and can reduce PARPi cytotoxicity [41]. Our analysis identified nine chromatin-associated genes, which are grouped below according to their established molecular functions.

#### 2.3.1. ATP-Dependent Chromatin Remodeling: ACTL6A, ARID2, and CHD8

ATP-dependent chromatin remodelers reposition or evict nucleosomes and thereby regulate DNA accessibility during transcription and repair. SWI/SNF-family complexes participate in chromatin remodeling around double-strand breaks, facilitating BRCA1 recruitment and homologous recombination [63].

*ACTL6A* contributes to complex organization and regulates SWI/SNF engagement with chromatin [64]. This gene is unvalidated in the PARPi response context and hypothetically its loss may alter chromatin remodeling by reducing trapping-associated toxicity. CHD8 binds to p53 and limits its pro-apoptotic activity [65]. However, the role of CHD8 in the response to PARPi has not been investigated.

*ARID2* loss impaired DNA repair and sensitized lung cancer cells to PARPi [66]. Another study demonstrated that deficiency of another SWI/SNF-family subunit, ARID1A, led to PARPi sensitization [67]. These findings do not explain the resistance-associated enrichment of ARID2 in our analysis. We hypothesize that ARID2 loss in some cellular backgrounds may reduce PARP1 chromatin retention, alter cell cycle progression, and decrease PARPi toxicity.

#### 2.3.2. Histone Methylation and Active-Chromatin Recognition: WDR5, CARM1, and PHIP

Methylation-dependent chromatin states are linked to H3K4 and arginine methylation and it maintains transcriptionally active chromatin and regulates the expression of DDR genes. WDR5 interacts with PARP1 to facilitate target-chromatin binding, and combined WDR5 WIN-site and PARP inhibition suppresses proliferation [68]. CARM1 methylates the SWI/SNF subunit BAF155 and alters its chromatin localization, which links CARM1 to transcriptional regulation and tumor progression [69]. CARM1 promotes PARP1 association and PARylation at replication forks through a methyltransferase-independent function [70]. CARM1 loss reduces fork reversal and PARPi sensitivity. We propose that reduced PARP1 trapping may contribute to PARPi resistance associated with CARM1 loss. PHIP, a chromatin reader that integrates methylation and acetylation signals, recruits CRL4 ubiquitin ligases to chromatin [71]. Its loss could therefore alter protein turnover at active chromatin or DNA-damage sites. However, a direct role for PHIP in PARPi response has not yet been established.

#### 2.3.3. The Acetylation-Coactivator Axis: YY1, KAT7, and BRD1

Histone acetyltransferases neutralize the positive charge of histone lysine residues, weakening their contact with DNA and opening chromatin for transcription and DNA repair. KAT7 and BRD1 emerged from our screen analysis, whereas YY1 is discussed here based on published evidence of PARP1 regulation [72]. YY1 is a transcription factor that binds the PARP1 promoter and stimulates its PARylation [72]. These findings establish a link between YY1 and PARP1 regulation, but the role of YY1 in PARPi response should be investigated further. KAT7 (HBO1) is a histone acetyltransferase whose substrate depends on associated subunits [73]. BRD1 (BRPF2) is a scaffold that bridges HBO1 and ING4 and supports H3K14 acetylation [74]. As a result of our analysis, transcriptional and chromatin changes influence PARPi response, but their involvement in PARPi response remains unvalidated.

### 2.4. Post-Translational Regulation

Genes acting after protein synthesis formed a smaller but consistently represented category in our analysis. This resistance module includes deubiquitinating enzymes, enzymes of the hexosamine and N-linked glycosylation pathways, and components of ER-to-Golgi vesicular transport. Ubiquitin marks placed on histone H2A at damaged chromatin determine which repair factors are recruited, while glycosylation and trafficking control how stress-response and survival proteins are processed and where they accumulate. Loss of these genes may therefore change PARPi response indirectly, by altering repair signaling or the handling of proteins that support survival under replication stress.

#### 2.4.1. The Ubiquitin/Deubiquitinating Enzyme Axis: USP3, USP16, USP28, and USP48

The ubiquitin system is a major regulator of the DNA-damage response. At sites of double-strand breaks, E3 ubiquitin ligases modify histone H2A and create binding platforms for downstream repair factors, including TP53BP1 [75]. Deubiquitinating enzymes (DUBs) counterbalance these signals by removing ubiquitin marks and thereby modulating DNA-repair pathway activity. Several DUBs emerged as PARPi-response-associated hits, including *USP3*, *USP16*, *USP28*, and *USP48*. Among them, USP28 has the strongest mechanistic link to drug response. Pharmacological inhibition of USP28 has been reported to destabilize SOX9, impair DNA repair, and increase PARPi sensitivity in preclinical models [76]. In our integrated analysis, however, *USP28* knockout was associated with PARPi resistance. This discrepancy suggests that the effect of USP28 may depend on cellular background, baseline repair capacity, or the mode of perturbation. Further validation is required to determine whether USP28 can exert bidirectional effects on PARPi response.

USP3, USP16, and USP48 also regulate histone H2A ubiquitylation and DNA-repair signaling [77,78,79]. USP48, for example, limits DNA-end resection by modulating BRCA1–BARD1-associated ubiquitin marks. Loss of USP48 can therefore increase end resection and alter double-strand break repair-pathway choice [77]. Together, these findings support a role for DUBs in shaping PARPi response, although the direction and magnitude of their effects appear to be strongly context-dependent.

#### 2.4.2. Glycometabolic Flux and Protein Glycosylation: GFPT1, DPAGT1

The identification of *GFPT1*, a rate-limiting enzyme in the hexosamine biosynthetic pathway, and *DPAGT1*, which catalyzes an early step in N-linked glycosylation, points to a potential metabolic and post-translational layer of PARPi resistance. These enzymes connect glucose and glutamine metabolism to protein glycosylation [80]. Altered glycosylation could influence the stability, localization, or signaling of growth-factor receptors and other stress-response proteins, thereby modifying cellular tolerance to PARPi. However, a direct mechanistic link to PARPi resistance remains to be established.

#### 2.4.3. Vesicular Trafficking and Protein Localization: TMED2, COG4

Vesicular trafficking proteins, including *TMED2* and *COG4*, regulate transport between the endoplasmic reticulum and Golgi through COPI- and COPII-associated pathways [81,82,83]. Their identification as resistance-associated hits may reflect altered processing or localization of receptors and other proteins involved in survival signaling. At present, however, their specific contribution to PARPi resistance remains unclear and requires direct functional validation.

### 2.5. Mitochondrial Function

Beyond canonical DNA-repair mechanisms, cellular metabolism has emerged as a potential determinant of PARPi response. PARP1 consumes NAD^+^ during PARylation, linking its activity to the cellular metabolic state. Mitochondria contribute to NAD^+^/NADH balance, ATP production, and redox homeostasis. DNA damage leads to PARP1 activation, which can deplete NAD^+^ and increase cellular dependence on oxidative phosphorylation for survival [84]. Therefore, PARP1 is linked to mitochondrial metabolism, although the contribution of this metabolic response to PARPi resistance remains to be established.

**Figure 2:**
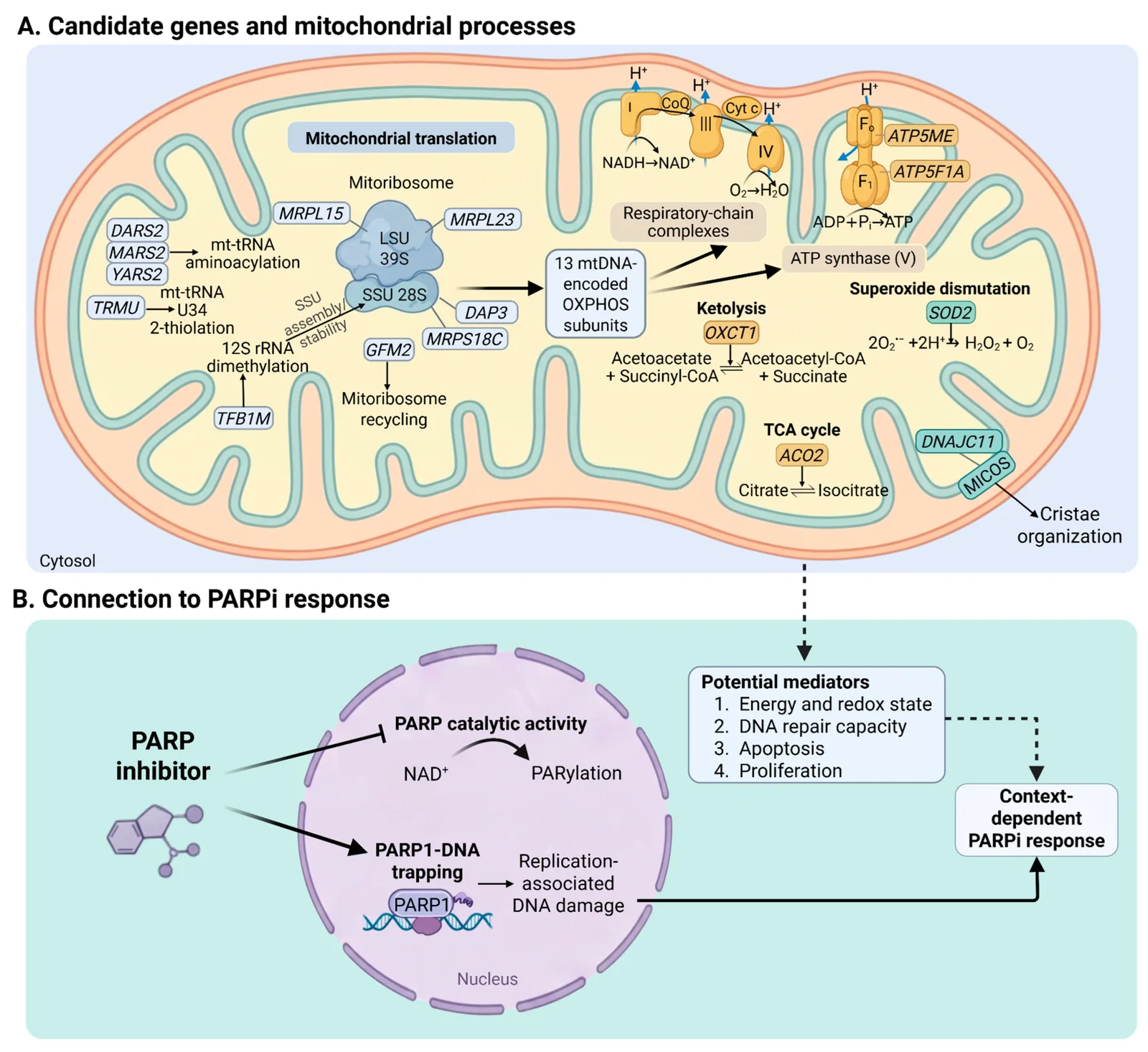
Mitochondrial modulation of PARPi response. (**A**) Candidate genes identified in the cross-screen analysis are mapped to mitochondrial translation, energy metabolism, redox regulation and cristae organization. The diagram shows their involvement in OXPHOS, the TCA cycle, ketolysis, superoxide dismutation and cristae organization. Gene-label colors indicate functional groups: mitochondrial translation (light blue), energy metabolism (yellow), and antioxidant defense and cristae organization (green). (**B**) PARPi suppress PARP catalytic activity and promote PARP1–DNA trapping, contributing to replication-associated DNA damage. Mitochondrial processes may modify PARPi response through changes in energy and redox state, DNA repair capacity, apoptosis and proliferation. Dashed arrows indicate proposed functional links between mitochondrial processes and PARPi response. Abbreviations: SSU, small mitoribosomal subunit (28S); LSU, large mitoribosomal subunit (39S); mtDNA, mitochondrial DNA; mt-tRNA, mitochondrial transfer RNA; rRNA, ribosomal RNA; CoQ, coenzyme Q; Cyt c, cytochrome c; $F_{o}$, membrane-embedded proton-translocating sector of ATP synthase; $F_{1}$, matrix-facing catalytic sector of ATP synthase; PDH, pyruvate dehydrogenase; α-KGDH, α-ketoglutarate dehydrogenase; MICOS, mitochondrial contact site and cristae organizing system. Created in BioRender. Lezina, L. (2026) https://BioRender.com/m6hb01i, accessed on 16 October 2026.

Moreover, cells with mitochondrial dysfunction shift towards glycolysis, which can reduce olaparib sensitivity [85]. Figure 2 summarizes the functions of the mitochondrial candidate genes identified in our analysis and their potential links to PARPi response.

#### 2.5.1. Mitochondrial Translation: MRPL15, MRPL23, MRPS18C, DAP3, GFM2, DARS2, MARS2, YARS2, TRMU, and TFB1M

The mitochondrial translation group includes *MRPL15*, *MRPL23*, *MRPS18C*, *DAP3*, *GFM2*, *DARS2*, *MARS2*, *YARS2*, *TRMU*. The mitoribosome translates 13 mtDNA-encoded OXPHOS subunits, and impaired mitotranslation reduces respiratory-chain function [86].

MRPL15 and MRPL23 are subunits of the large (39S) mitoribosomal subunit, whereas MRPS18C and DAP3 belong to the small (28S) subunit. GFM2 is a mitochondrial elongation factor required for ribosome recycling after termination [87]. DARS2, MARS2, and YARS2 are mitochondrial aminoacyl-tRNA synthetases that load the corresponding amino acids onto mt-tRNA [88]. TRMU is a mitochondrial tRNA U34-modifying enzyme, which is necessary for decoding accuracy [88]. TFB1M catalyzes adenine dimethylation in mitochondrial 12S rRNA that supports small-subunit stability and mitochondrial translation [89].

DAP3, in addition to its structural role in the mitoribosome, has also been linked to proapoptotic signaling [90]. Impairments in mitochondrial function and a reduced response to apoptosis inducers have been identified in fibroblasts with biallelic DAP3 variants [91]. Therefore, these findings suggest that DAP3 dysfunction can affect mitochondrial protein synthesis and apoptosis, but involvement of these effects in PARPi resistance remains unresolved.

A recent study described a direct interaction of MRPL21, a related subunit of the mitoribosome, with PARP1, linking mitochondrial function to PARylation [92]. Although there is still little direct functional confirmation in the PARPi context, these findings collectively suggest a paradigm in which decreased mitochondrial translation may mitigate PARPi toxicity through altered OXPHOS and NAD^+^ homeostasis.

#### 2.5.2. OXPHOS and Energy Metabolism: ATP5A1, ATP5I, ACO2, and OXCT1

Another group of resistance hits includes OXPHOS and mitochondrial metabolic genes, notably *ATP5A1*, *ATP5I*, and *ACO2* that were identified as resistance hits in our analysis. ATP5A1 (ATP5F1A) and ATP5I (ATP5ME) are components of F_1_F_0_-ATP synthase (Complex V), with ATP5I additionally contributing to ATP synthase dimerization and cristae organization. ATP5I depletion disrupts ATP synthase organization, which leads to reduced mitochondrial respiration and increased lactate secretion [93]. Phosphorylation of ATP5F1A stabilizes complex V and increases mitochondrial energy output [94].

ACO2 catalyzes the conversion of citrate to isocitrate in the TCA cycle. Overexpression of ACO2 increases mitochondrial oxidation of pyruvate, reduces the glycolytic phenotype, and inhibits proliferation [95]. Previously it was observed that a shift towards glycolytic metabolism is associated with reduced olaparib sensitivity [85].

OXCT1 was not identified as a recurrent hit in our reanalysis but is discussed here because published findings link it to olaparib response [96,97]. OXCT1 is succinyl-CoA:3-oxoacid transferase, a key enzyme in the utilization of ketone bodies, an alternative source of acetyl-CoA [96]. In addition to its metabolic function, OXCT1 is involved in the regulation of RAD51. OXCT1-dependent succinylation of RAD51 impairs its binding to BRCA2 and suppresses HR [97]. In breast cancer models, increased RAD51 succinylation levels were associated with greater sensitivity to olaparib. Loss of OXCT1 may influence the response to PARP inhibitors by altering RAD51 function. However, this mechanism remains to be validated in the cell models used in the screens under analysis.

#### 2.5.3. Oxidative Stress and Mitochondrial Integrity: SOD2, KEAP1, and DNAJC11

Genes that belong to oxidative stress and mitochondrial integrity groups are *SOD2*, *KEAP1*, and *DNAJC11*. DNAJC11 is a mitochondrial co-chaperone involved in cristae formation through interaction with the MICOS complex [98]. SOD2 converts mitochondrial superoxide into hydrogen peroxide, which contributes to redox homeostasis [99]. Sod2 deficiency in a mouse model impaired mitochondrial complex II activity, which causes nuclear DNA damage [100]. Therefore, SOD2 regulates genome integrity and its role in PARPi response requires direct investigation.

KEAP1 promotes NRF2 ubiquitination and degradation, and KEAP1 loss can activate antioxidant defense programs that promote ROS detoxification [101]. Although KEAP1 is not a mitochondrial structural protein, it controls NRF2-dependent antioxidant buffering, a process that directly influences mitochondrial ROS stress and survival under PARPi. Given that PARPi-induced replication stress increases ROS production, activation of NRF2 upon loss of KEAP1 may provide a protective antioxidant response that reduces PARPi cytotoxicity.

The mitochondrial module identified in our analysis indicates that metabolic state can influence PARPi resistance alongside DNA repair, apoptosis and proliferation. Potential mechanisms include changes in NAD^+^ homeostasis, energy metabolism, antioxidant responses and apoptotic priming. Importantly, the majority of these genes remain screen-level observations and need to be investigated further. However, their recurrent identification across screens suggests that mitochondrial rewiring is an understudied and biologically plausible axis of PARPi adaptation.

### 2.6. TGF-β and mTOR Signaling

The TGF-β/SMAD and PI3K/AKT/mTOR pathways are linked to proliferation, epithelial–mesenchymal transition (EMT), and immune evasion, but their effects on PARPi response depend on cellular context.

#### 2.6.1. TGF-β/SMAD: TGFBR2, SMAD4, and FURIN

TGF-β/SMAD signaling modulates PARPi response in a highly context-dependent manner, with opposite effects across different tumor models. In leukemia models, stromal TGF-β1 under hypoxia activates the TGFβR-SMAD2/3 axis and enhances DSB repair, which protects tumor cells from PARPi-induced lethality [102]. Conversely, in breast cancer models, TGF-β reduced BRCA1, ATM, and MSH2 expression and increased sensitivity to veliparib [103]. *SMAD4*-deficient head and neck squamous cell carcinoma (HNSCC) models show increased sensitivity to olaparib in combination with radiotherapy [104]. These studies provide direct functional evidence for TGFBR2 and SMAD4 in PARPi response.

FURIN is involved in the proteolytic maturation of cancer-relevant substrates such as TGF-β-related ligands and growth factor receptors [105,106]. Its resistance phenotype could involve TGF-β-dependent effects, which require further research.

#### 2.6.2. mTOR: RPTOR

RPTOR (Raptor) is an essential scaffold component of mTORC1. PI3K inhibition reduced BRCA1/2 expression and sensitized breast cancer models to PARPi [107]. Moreover, mTOR inhibition impaired HR and enhanced PARPi activity [108]. This provides the rationale for combining PI3Ki or mTOR inhibitors with PARP inhibitors. Regarding clinical findings, alpelisib plus olaparib showed preliminary activity in ovarian cancer [109], whereas ipatasertib plus rucaparib did not demonstrate additive or synergistic antitumor activity in previously treated metastatic castration-resistant prostate cancer [110].

RPTOR enrichment in resistance screens may reflect changes in proliferation or adaptation to reduced mTORC1 activity. These possibilities require gene-specific validation with controls for growth rate and should be distinguished from the effects of pharmacological PI3K, AKT, or mTOR inhibition.

### 2.7. RNA Processing and Nucleocytoplasmic Transport

Genes controlling RNA fate and nuclear transport also recurred across independent screens in our analysis. This resistance module includes the m^6^A methyltransferase *METTL3*, subunits of the CCR4–NOT mRNA decay complex, a ribosome-associated quality-control factor, and several nucleoporins. Repair proteins such as BRCA1 and RAD51 must be transcribed, stabilized, translated, and imported into the nucleus before they can act at a damaged fork. Loss of RNA-processing or nuclear-pore components could therefore limit the supply of repair and checkpoint factors at the point where they are most needed.

#### 2.7.1. Epitranscriptomic Regulation and mRNA Stability

CRISPR screens identified several genes involved in mRNA processing and turnover. *METTL3*, the catalytic component of the m^6^A methyltransferase complex, appeared as a resistance-associated hit. m^6^A modification regulates mRNA stability and translational efficiency and has established roles in cellular stress responses [111]. Its identification therefore raises the possibility that altered epitranscriptomic regulation changes the expression dynamics of DDR or replication-stress genes. However, the mechanism by which *METTL3* loss might confer PARPi resistance has not been established.

Components of the CCR4–NOT mRNA decay complex, including *CNOT4* and *CNOT11*, were also identified, together with the ribosome-associated quality-control factor *HBS1L* [112,113]. These findings suggest that mRNA turnover and translational quality control may contribute to adaptation under PARPi-induced stress, although direct mechanistic evidence remains limited.

#### 2.7.2. The Nuclear Pore Complex and Nucleocytoplasmic Transport

Several components of the nuclear pore complex, including *NUP107, NUP85, NUP155,* and *NUP43*, were identified as recurrent hits [114,115,116]. The nuclear pore complex (NPC) regulates the bidirectional transport of proteins and RNA between the nucleus and cytoplasm, and its disruption can alter the nuclear availability of DNA-repair factors [117].

Because HR proteins such as BRCA1 and RAD51 depend on regulated nuclear trafficking, NPC perturbation could influence HR efficiency during PARPi-induced replication stress. NPC dysfunction may also affect the transport of transcripts involved in checkpoint control, including *CDKN1A* (p21), thereby modifying cellular responses to DNA damage [118].

We therefore hypothesize that disruption of specific nucleoporins may alter the nuclear availability of HR and checkpoint factors and thereby modify PARPi response. This mechanism remains speculative and requires direct functional validation.

### 2.8. Emerging Adaptive Mechanisms

A final group of recurrent genes was not mapped to any of the seven modules above by Reactome enrichment analysis. These hits are functionally mixed and include the replication-stress kinases *TLK2* and *CDK18*, the stress-activated kinases *MAP4K2* and *MAP4K3*, and a set of ubiquitin, phosphatase, RNA-binding, and cytoskeletal regulators with no established link to PARP inhibition. What these genes share is recurrence across independent screens rather than membership in a common pathway. We therefore treat this module as hypothesis-generating and focus on the candidates with the clearest connection to replication-stress biology.

Among these, *TLK2* is one of the most mechanistically plausible candidates. TLK2 regulates the histone chaperone ASF1 and supports replication-coupled chromatin reassembly behind the replication fork. Its loss increases baseline replication stress [119]. In the context of PARPi treatment, TLK2 disruption could alter chromatin organization at stalled forks and thereby modify the consequences of PARP1 trapping, although this has not been directly tested.

*CDK18* is a cyclin-dependent kinase linked to ATR signaling during replication stress [120,121]. Its loss may alter checkpoint activation and S-phase progression under PARPi-induced stress. A possible consequence is increased tolerance of unresolved replication-associated damage, but this mechanism remains hypothetical.

*MAP4K2* and *MAP4K3* point to a broader stress-signaling axis. Both can feed into JNK and p38 MAPK pathways, which regulate stress responses and apoptosis. Their loss may therefore alter the threshold for damage-induced cell death and permit survival under increased genotoxic stress [122].

Other recurrent hits are less well characterized in this setting. *RNF10*, a RING-domain E3 ubiquitin ligase, and *PTP4A1*, an oncogenic phosphatase, have established roles in cancer-cell signaling and survival but have not been directly linked to PARPi resistance [123,124]. *MATR3* encodes a nuclear matrix protein involved in RNA processing and nuclear organization and may connect this module to the post-transcriptional mechanisms discussed in Section 2.7 [125]. *ACVR1*, a BMP type I receptor with oncogenic signaling functions, and *INF2*, a formin involved in cytoskeletal dynamics and nuclear-envelope organization, remain more speculative candidates in the PARPi context [126,127,128].

Overall, *TLK2* and *CDK18* provide the strongest starting points for functional follow-up because of their established links to replication-stress biology. The remaining genes should currently be regarded as hypothesis-generating candidates that require direct validation in PARPi-treated models.

## 3. Discussion

### 3.1. Interpretation of Recurrent Hits

Reanalysis of published CRISPR knockout screens identified recurrent olaparib-response candidates across DNA repair, cell-cycle control, chromatin regulation and mitochondrial processes. Comparing screens helps identify candidates supported by more than one experiment and places less characterized genes alongside established determinants such as PARP1 and TP53BP1 [13,22]. Recurrence provides a basis for prioritization but does not establish the mechanism underlying the screening phenotype. For candidates such as MRPL15, CNOT4 and NUP107, the proposed links to olaparib resistance are based mainly on their recurrence across screens and their known biological functions. Whether these functions explain the resistance phenotype still needs to be experimentally validated.

Our main contribution is an olaparib-focused comparison of selected screens using standardized reanalysis and prioritization of recurrent candidates. This adds a focused comparison of olaparib CRISPR knockout screens to previous reviews and broader cross-screen analysis [9,24,32]. For mitochondrial genes, we identify recurrent candidates across screens and compare their screening phenotypes with published mechanistic evidence [85,97].

Mitochondrial candidates warrant particular attention because they span protein synthesis, respiratory and TCA-cycle metabolism, redox regulation and cristae organization. Published studies already connect metabolic state to PARPi response [85]. OXCT1-dependent RAD51 succinylation also links a metabolic enzyme to HR regulation and olaparib sensitivity [97]. The contribution of the present analysis is therefore to identify recurrent candidates within these processes. Their shared annotation does not imply a uniform effect of mitochondrial dysfunction, and altered mitochondrial metabolism should not be assumed to deplete the NAD^+^ pool available to nuclear PARP1.

Discordance between screen enrichment and published functional evidence is another important finding. Genes such as *PPP2R2A, RB1, SMAD4, FURIN,* and *CARM1* may have different, or even opposing, effects depending on HR status, tumor lineage, and treatment context. PPP2R2A loss has produced different PARPi responses in lung and prostate cancer models [33,50], whereas ARID2 depletion sensitized lung cancer cells despite it being observed as a resistance hit in our analysis [66]. Such discrepancies may reflect genetic background, baseline proliferation, drug exposure or differences between genetic and pharmacological perturbations. CRISPR screens therefore provide a powerful map of candidate resistance determinants, but their interpretation requires careful consideration of the underlying biological context.

### 3.2. Future Directions

Follow-up should first establish whether individual candidates from our analysis can show the same resistance phenotype to olaparib using independent perturbations, genetic rescue, untreated growth controls and long-term survival assays. Testing more than one genetic background would help define the scope of each phenotype. For mitochondrial candidates, mechanistic measurements should be selected according to gene function and may include mitochondrial translation, respiration, compartment-specific NAD^+^ homeostasis, HR activity or apoptotic responses. These experiments would distinguish direct effects on damage handling from changes in proliferation or short-term survival. Combination studies should follow confirmation of the direction of the phenotype, because inhibiting a gene whose loss promotes resistance could reduce PARPi activity.

### 3.3. Limitations

The main limitation is the substantial heterogeneity across the analyzed CRISPR screens. The primary datasets were generated in diverse cell-line backgrounds that differ in homologous recombination capacity, *BRCA1/2* status, p53 function, and baseline genomic instability. Experimental parameters also varied considerably, including CRISPR library design, guide representation, drug concentration, and treatment duration. For example, Lin et al. used an IC20-based treatment condition, whereas Lau et al. initially employed an IC90–99-based selection strategy. In the available information for Lin et al., the treatment concentration was reported as IC20 without an absolute molar concentration. These differences are likely to influence selective pressure and may explain why some genes emerge as resistance hits only in specific contexts.

A second limitation is that the analysis is olaparib-centric, therefore gene sets identified here should not be assumed to represent universal determinants of resistance across the entire PARPi class. PARP inhibitors differ substantially in catalytic inhibition, PARP-trapping potency, pharmacologic properties, and clinical use. The relative importance of individual resistance mechanisms may therefore vary between compounds.

An additional limitation is that the present study is based on the computational reanalysis of previously published screening datasets, without independent experimental validation of the identified candidates. Although recurrence across screening models provides evidence for reproducible resistance-associated phenotypes, it does not establish the molecular mechanisms underlying these observations. This distinction is particularly important for candidates associated with mitochondrial metabolism, RNA processing, and other less-characterized pathways. The proposed mechanisms should therefore be regarded as hypotheses requiring validation through targeted genetic perturbation, rescue experiments, and functional assays in appropriate cellular models.

Finally, our hit-selection strategy incorporated bidirectional information from resistance and sensitivity analyses. In studies designed primarily to identify sensitivity, we also considered genes from resistance-oriented analyses when they met the predefined statistical threshold for resistance (FDR < 0.05). Consequently, some recurrent hits may partly reflect differences in screen design rather than stable resistance mechanisms and require independent functional validation.

## 4. Materials and Methods

### 4.1. The Identification of CRISPR Screens

A search for published CRISPR-Cas9 screens using PARP inhibitors was conducted in the BioGRID ORCS database: https://orcs.thebiogrid.org/Search?searchType=key&search=olaparib+&organism=9606 (accessed on 23 February 2026). We identified 68 screening records using the keyword “olaparib” and restricting the organism to Homo sapiens. As individual publications could contribute multiple records corresponding to different cell models, libraries, or experimental conditions, records were consolidated according to their originating publication. This yielded 12 distinct publications for eligibility assessment. For each screen, the completeness of the experimental design and the availability of raw data were assessed. Two publications were excluded at this stage because there were no raw data or FASTQ files even upon request [129,130]. Of the 10 publications meeting eligibility criteria, raw guide RNA count tables or FASTQ files were already publicly available for 8 publications. For the remaining 2 publications, count tables were requested from the corresponding authors by email. In total, 10 publications met all inclusion criteria and were carried forward for reanalysis. The details of the search on BioGRID ORCS are shown in Figure S1. Identified, excluded, and included screens, with reasons for exclusion, are listed in Table S3.

### 4.2. Enrichment and Depletion Analysis

To standardize the analysis, all count tables were processed with MAGeCK (version 0.5.9.5). Robust rank aggregation (RRA) and maximum-likelihood estimation (MLE) were performed using the “mageck test” and “mageck mle” commands, respectively. Scripts for MAGeCK RRA and MLE analyses are available on GitHub: https://github.com/diana-samarkhanova/Olaparib-CRISPRscreen-reanalysis (accessed on 16 October 2026). sgRNA read counts were normalized using the median normalization method. For the reanalysis we compared olaparib-treated samples with the corresponding DMSO-treated controls within each study. Replicate samples were retained as separate columns in the sgRNA count matrix. For these datasets, genes with a positive-selection false discovery rate (FDR) < 0.05 in RRA or a positive β coefficient (treatment-versus-control differential β-score) and FDR < 0.05 in MLE were considered resistance-associated screening hits. Genes identified exclusively through negative selection (sensitization) were excluded from the recurrence analysis.

For recurrence analysis, an individual screening unit was defined as a distinct cell-line screen within an included publication. Screens performed in different cellular models were counted separately, even when reported in the same study. In contrast, multiple treatment time points from the same screening experiment were combined and counted once per gene. Thus, repeated identification of a gene across time points or complementary libraries did not increase its recurrence count. A gene was classified as recurrent when it was positively selected in at least two screening units. The contributing publications, cell models, screening identifiers, and available gene-level statistical results are provided in Supplementary Table S1.

### 4.3. Analysis and Pathway Enrichment

Genes identified as resistance hits in two or more independent screens (*n* = 163) were selected for further analysis. The details of each gene are listed in Table S1. This list was subjected to pathway enrichment analysis using the Reactome Pathway Analysis service (release 96) for Homo sapiens, which identified 983 pathways, of which 329 had FDR < 0.05 (Table S2). The enriched pathways were grouped into seven broader functional modules according to their biological functions. Genes that were recognized by Reactome but were not represented in the significantly enriched pathways used for these modules were examined separately. Based on their reported biological functions and published literature, these genes were classified into an eighth group “Emerging Adaptive Mechanisms”. Because individual genes could be associated with multiple significantly enriched Reactome pathways and biological processes, each recurrent gene was assigned to one primary functional module based on its predominant literature-supported function. Additional functional associations were documented separately without duplicating genes across modules. The complete gene-to-module assignments, supporting Reactome pathways, and assignment notes are provided in Supplementary Table S2.

## 5. Conclusions

Reanalysis of published CRISPR knockout screens identified candidate determinants of PARPi response beyond DNA repair, including cell-cycle control, chromatin regulation and mitochondrial metabolism. These findings support a broader view in which PARPi resistance reflects coordinated adaptation across multiple cellular systems rather than restoration of a single pathway.

Mitochondrial genes involved in translation, energy metabolism and redox regulation warrant particular attention. Their recurrence across screens supports further investigation of metabolic contributions to PARPi response, although the mechanisms remain unresolved for many candidates. Follow-up studies should establish how individual gene perturbations affect drug response and distinguish durable resistance from effects on proliferation or short-term survival.

## Figures and Tables

**Table 1:** Characteristics of published CRISPR screening studies investigating olaparib response.

Study	Cellular Model/Cancer Type	Cell Line (s)	CRISPR Library	Olaparib Treatment Conditions	Original Analysis Method	Key Findings
Zimmermann et al., 2018 [27]	Cervical/retinal pigment epithelium/triple-negative breast cancer	HeLa; RPE1-hTERT; SUM149PT	TKOv1, genome-wide CRISPR-KO	HeLa: 2 µM; RPE1: 0.5 µM; SUM149PT: 0.2 µM; 15 days	DrugZ, MAGeCK	RNASEH2A/B/C identified as top sensitizers; PARP1 associated with resistance in RER-deficient cells
Juhász et al., 2020 [30]	Cervical, Osteosarcoma	HeLa	Brunello, genome-wide CRISPR-KO	3 µM, 14 days	DrugZ	ALC1/CHD1L identified as a PAR-dependent chromatin remodeler whose loss enhances PARPi sensitivity
Fang et al., 2019 [28]	Ovarian	A2780	GeCKOv2 (A and B), genome-wide	5 and10 µM, 7 days	MAGeCK	C12orf5/TIGAR identified as a sensitizer; associated with increased ROS and induction of a BRCAness phenotype
Clements et al., 2020 [29]	Cervical	HeLa-BRCA2-KO	Brunello, genome-wide CRISPR-KO; Calabrese, genome-wide CRISPRa	4 µM, 4 days	MAGeCK	HUWE1 associated with resistance through increased RAD51; KAT5/TIP60 associated with resistance through 53BP1 promotion in BRCA2-deficient cells
Olivieri et al., 2020 [32]	Immortalized human retinal pigment epithelium (RPE1)	RPE1-hTERT p53−/−	TKOv3, genome-wide CRISPR-KO	5 µM, 18 days	DrugZ	Genetic map of 890 DNA damage response genes; identified olaparib-associated hits across a 31-screen dataset
Lau et al., 2020 [31]	Lymphoma/leukemia	HAP1	GeCKO v2, genome-wide CRISPR-KO	25 µM, 3 days	MAGeCK	No significant olaparib hits were identified, representing an important negative finding; 26 other drugs produced significant hits
Zelceski et al., 2023 [35]	Breast (non-tumorigenic)	MCF10A TP53−/−	Yusa CRISPR-KO genome-wide; Weissman CRISPRi-v2	2.5 µM, 14 days	MAGeCK Z-score	MND1 and PSMC3IP identified as potent sensitizers; both encode components of a meiotic recombination complex
Tuano et al., 2023 [34]	Breast (immortalized mammary epithelial cells)	HMLE, mesHMLE, B80-T5, B80-T17, K5+/K19−	Custom targeted CRISPR-KO/i/a libraries	2.5–5 µM, 14 days	MAGeCK	Identified 28 breast cancer risk-associated DDR genes; SIVA1 and CMTR2 emerged as novel sensitizers
Lin et al., 2024 [36]	Non-tumorigenic epithelial	RPE1 TP53−/−; HAP1 (comparison screen)	Custom DDR-focused library, TKOv3 CRISPR-KO genome-wide	IC20, 18 days	Adapted Orthrus pipeline; CGI score	Validated HR pathway hits and revealed late engagement of the interstrand cross-link repair pathway
Tsujino et al., 2023 [33]	Prostate cancer	LNCaP; C4-2B; 22Rv1; DU145	H1 + H2, genome-wide	5–10 µM, 28 days	MAGeCK	MMS22L associated with sensitization in a TP53-dependent manner; CHEK2 associated with resistance through increased BRCA2; PPP2R2A associated with resistance

**Note:** The table summarizes the experimental designs and principal findings reported in the original publications. Only CRISPR-Cas9 knockout datasets for which suitable sgRNA count tables or raw sequencing data were available were analyzed. Specifically, CRISPRi and CRISPRa datasets were excluded, as well as cellular models for which the required count data were unavailable. The individual datasets included in our analysis, together with their treatment conditions, analytical screening units, and inclusion criteria, are detailed in Supplementary Table S3. The original screening analyses are reported in Table 1, whereas the standardized MAGeCK-RRA and MAGeCK-MLE reanalysis is described in Section 4.2. Multiple time points or olaparib concentrations within the same screening series were counted as one analytical unit for recurrence analysis. DDR, DNA damage response; HR, homologous recombination; MAGeCK, Model-based Analysis of Genome-wide CRISPR-Cas9 Knockout; PARPi, PARP inhibitor; CRISPR-KO, CRISPR knockout; CRISPRi, CRISPR interference; CRISPRa, CRISPR activation; CGI, chemical-genetic interaction; TKO, Toronto KnockOut library.

**Table 2:** Core biological modules underlying PARP inhibitor resistance identified by CRISPR Screen.

Functional Module	Core Pathway	Representative Genes	Proposed Mechanism of PARPi Resistance
DNA damage repair & replication stress	PARP trapping; HR pathway choice; fork protection	*PARP1* ^a^, *PARG* ^b^, *ADPRHL2* ^a^, *TP53BP1* ^a^, *PARP2*, *PRKDC* ^b^, *BRIP1* ^c^, *MYC*, *RRM2* ^†^	Reduced PARP trapping toxicity or restored HR via end-resection; NHEJ bias or enhanced nucleotide supply mitigates replication stress
Cell cycle regulation	G1/S & G2/M checkpoints; CDK–RB–E2F signaling; mitotic control	*PPP2R2A* ^†^, *TP53*, *CHEK2* ^b^, *CDK2* ^†^, *RB1* ^b^, *TPX2* ^c†^, *TAOK1* ^c†^, *E2F3* ^†^, *E2F7* ^b^, *HAUS3*	Loss of checkpoint control or altered S-phase entry reduces lethal replication stress and mitotic catastrophe
Chromatin remodeling & histone regulation	ATP-dependent chromatin remodeling; histone methylation and acetylation	*WDR5* ^c†^, *CARM1* ^c^, *ARID2* ^c†^, *ACTL6A*, *CHD8*, *PHIP*, *KAT7*, *BRD1*	Altered chromatin accessibility reduces PARP1 recruitment and destabilizes trapped complexes
Post-translational regulation	H2A deubiquitylation; glycosylation; trafficking	*USP48* ^c^, *USP28* ^c†^, *USP3*, *USP16*, *GFPT1*, *DPAGT1*, *TMED2*, *COG4*	DUB and glycosylation rewiring alters DSB scaffolds and activates compensatory survival signaling
Mitochondrial function	Mitochondrial translation; respiratory & TCA metabolism; KEAP-NRF2 stress response	*DAP3* ^c^, *KEAP1* ^c^, *MRPL15*, *MRPL23*, *MRPS18C*, *DARS2*, *MARS2*, *YARS2*, *TRMU*, *GFM2*, *ATP5A1*, *ATP5I*, *ACO2*, *OXCT1* ^c†^, *TFB1M*, *SOD2*, *DNAJC11*	Mitochondrial translation, metabolic and redox functions, and cristae organization. NAD^+^/NADH balance and ROS
TGF-β & mTOR signaling	TGF-β receptor-SMAD signaling; mTORC1-dependent translation, autophagy	*RPTOR* ^†^, *SMAD4* ^c†^, *TGFBR2* ^c†^, *FURIN*	Microenvironment-driven HR enhancement and metabolic rewiring can promote survival under PARPi stress
RNA processing & Nucleocytoplasmic transport	m^6^A RNA regulation; NPC transport; mRNA decay	*METTL3*, *CNOT4*, *CNOT11*, *HBS1L*, *NUP107*, *NUP85*, *NUP155*, *NUP43*	Impaired RNA maturation/export reduces DDR protein availability and weakens replication stress response
Emerging Adaptive Mechanisms	ATR signaling; chromatin assembly; RNA & cytoskeletal regulation	*TLK2*, *CDK18* ^c^, *MAP4K2*, *MAP4K3*, *RNF10*, *PTP4A1*, *MATR3*, *ACVR1*, *INF2*	Putative adaptive rewiring of genome stability, apoptosis threshold, and nuclear architecture under PARPi pressure

**Note:** Superscripts describe proposed evidence status for the perturbation and phenotype under discussion, not a hierarchy based on number of pooled-screen occurrences: ^a^, targeted resistance phenotypes supported in ≥2 independent publications; ^b^, targeted resistance phenotype supported in one publication; ^c^, published functional or mechanistic connection to PARP inhibitor response without established knockout-mediated resistance validation. Genes without a superscript are screen-associated candidates for which independent phenotypic resistance validation has not been established by this audit. ^†^ Published sensitization, opposite-direction observations, or perturbation/context-dependent drug responses are also reported. These studies must not be counted as direct validation that gene loss confers olaparib resistance. For genes without direct functional tests, module-level mechanisms remain proposed rather than established. RRA/MLE recurrence is screening evidence, not independent experimental validation.

## Data Availability

No new experimental data were generated in this study. All CRISPR-Cas9 screen datasets reanalyzed here were obtained from the BioGRID Open Repository of CRISPR Screens (BioGRID ORCS, https://orcs.thebiogrid.org/Search?searchType=key&search=olaparib+&organism=9606, accessed on 16 October 2026). The raw sequencing files and sgRNA count tables used for reanalysis were obtained from publicly available data sources or provided by the corresponding authors of the original studies upon request.

## Supplementary Materials

The following supporting information can be downloaded at: https://www.mdpi.com/article/10.3390/ijms27209381/s1.

## Abbreviations

The following abbreviations are used in this manuscript:

PARP	Poly(ADP-ribose) polymerase
PARPi	PARP inhibitor
HR	Homologous recombination
HRR	Homologous recombination repair
NHEJ	Non-homologous end joining repair
DDR	DNA damage response
DSB	Double-strand break
SSB	Single-strand break
ssDNA	Single-stranded DNA
ROS	Reactive oxygen species
OXPHOS	Oxidative phosphorylation
MAGeCK	Model-based Analysis of Genome-wide CRISPR-Cas9 Knockout
FDR	False discovery rate
LFC	Log fold change
CGI	Chemical-genetic interaction
CRISPR	Clustered Regularly Interspaced Short Palindromic Repeats
Cas9	CRISPR-associated protein 9
CRISPR-KO	CRISPR Knockout
CRISPRa	CRISPR Activation
CRISPRi	CRISPR Interference
sgRNA	Single -guide RNA
BER	Base excision repair
ctDNA	Circulating tumour DNA
HRD	Homologous recombination deficiency
NPC	Nuclear Pore Complex
RRA	Robust Rank Aggregation
MLE	Maximum Likelihood Estimation
ORCS	Open Repository of CRISPR Screens
EMT	Epithelial-Mesenchymal transition
mTOR	Mechanistic target of rapamycin
TGF-β	Transforming growth factor beta
