# Supplementary material for: Beyond Homologous Recombination: Functional Modules of Olaparib Resistance Revealed by CRISPR Screens
Source: Int J Mol Sci. 2026 Oct 21;27(20):9381. doi: 10.3390/ijms27209381 (PMC13705357; doi:10.3390/ijms27209381)
Supplement: Supplementary file 1 [file ijms-27-09381-s001.zip › Figure S1_Flow diagram of the published CRISPR screening studies investigating olaparib response.pdf]

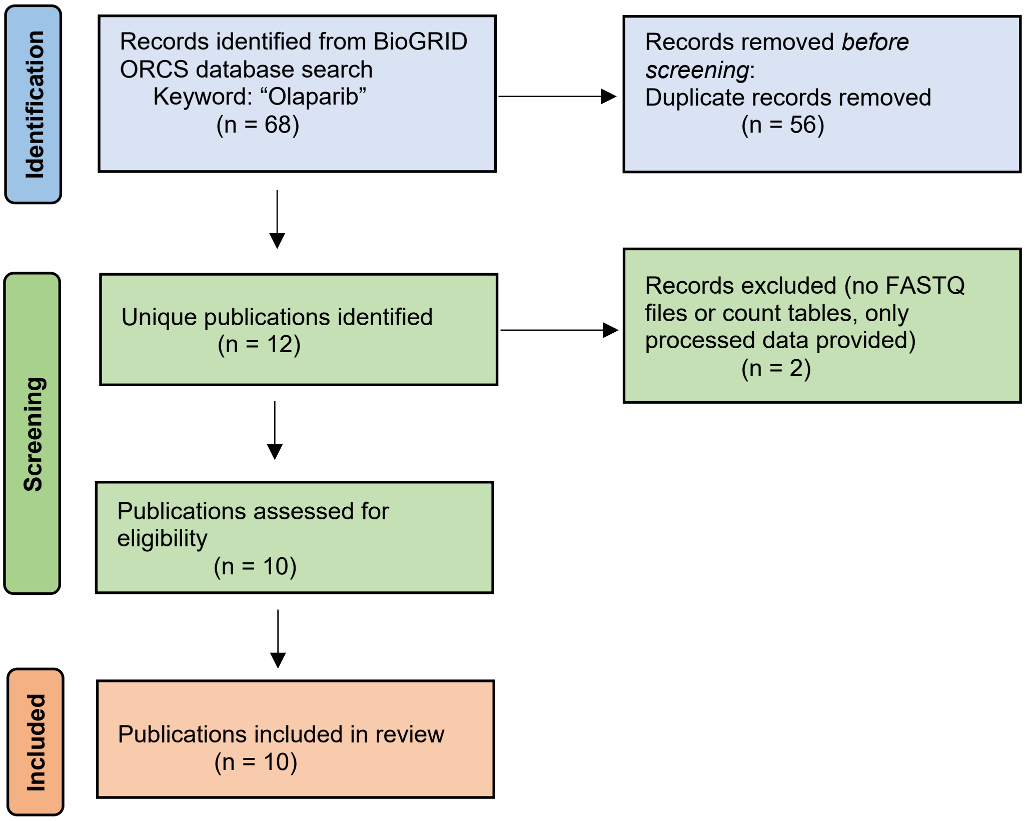

Figure S1. Flow diagram of the identification, eligibility assessment, and inclusion of published CRISPR-Cas9 screening studies investigating olaparib response. A search of the BioGRID Open Repository of CRISPR Screens (BioGRID ORCS) using the keyword "olaparib" and restricting the organism to *Homo sapiens* (NCBI Taxonomy ID: 9606) identified 68 screening records (https://orcs.thebiogrid.org/Search?searchType=key&search=olaparib&organism=9606). As individual publications could contribute multiple database records corresponding to different cell lines, screening libraries, or experimental conditions, records were consolidated according to their originating publication. This resulted in 12 distinct publications for eligibility assessment, with 56 additional records consolidated at the publication level. Each publication was assessed for the availability of raw sequencing files (FASTQ) or sgRNA count tables suitable for reanalysis. When these data were not publicly available, the corresponding authors were contacted. Two publications were excluded because the required data could not be obtained, leaving 10 publications eligible for inclusion. Within these publications, available CRISPR-Cas9 knockout datasets were selected for standardized reanalysis. The eligibility criteria, reasons for exclusion, and individual screening datasets included in the analysis are detailed in Supplementary Table S3.
